# A Novel Percutaneous Electrode Implant for Improving Robustness in Advanced Myoelectric Control

**DOI:** 10.3389/fnins.2016.00114

**Published:** 2016-03-31

**Authors:** Janne M. Hahne, Dario Farina, Ning Jiang, David Liebetanz

**Affiliations:** ^1^Institute of Neurorehabilitation Systems, University Medical Center GöttingenGöttingen, Germany; ^2^Department of Systems Design Engineering, Centre for Bioengineering and Biotechnology, University of WaterlooWaterloo, ON, Canada; ^3^Department of Clinical Neurophysiology, University Medical Center GöttingenGöttingen, Germany

**Keywords:** implant, electromyography, myoelectric control, regression, simultaneous control, prosthetic hand, robust control

## Abstract

Despite several decades of research, electrically powered hand and arm prostheses are still controlled with very simple algorithms that process the surface electromyogram (EMG) of remnant muscles to achieve control of one prosthetic function at a time. More advanced machine learning methods have shown promising results under laboratory conditions. However, limited robustness has largely prevented the transfer of these laboratory advances to clinical applications. In this paper, we introduce a novel percutaneous EMG electrode to be implanted chronically with the aim of improving the reliability of EMG detection in myoelectric control. The proposed electrode requires a minimally invasive procedure for its implantation, similar to a cosmetic micro-dermal implant. Moreover, being percutaneous, it does not require power and data telemetry modules. Four of these electrodes were chronically implanted in the forearm of an able-bodied human volunteer for testing their characteristics. The implants showed significantly lower impedance and greater robustness against mechanical interference than traditional surface EMG electrodes used for myoelectric control. Moreover, the EMG signals detected by the proposed systems allowed more stable control performance across sessions in different days than that achieved with classic EMG electrodes. In conclusion, the proposed implants may be a promising interface for clinically available prostheses.

## Introduction

Electromyography (EMG) has been used to control electrically powered hand and arm prostheses since decades (Parker et al., 2006; Farina et al., 2014). The techniques most often used in commercial prostheses are very simple and allow the control of only one degree of freedom (DoF) at a time. Typically the amplitudes of two bipolar surface EMG signals from antagonistic muscle-groups are subtracted to control one DoF in positive or negative direction. An extension of this approach, known as direct or differential control, is not possible with surface EMG, as usually not enough independent control sites are available. An exception consists of users with targeted muscle reinnervation (TMR), in which additional control sites are obtained by reinnervating the nerves that controlled the lost limb into other muscles (Kuiken, 2006).

To overcome the limitations of conventional control schemes, significant research efforts based on classification (Oskoei and Hu, 2007; Scheme and Englehart, 2011) and regression (Hahne et al., 2014; Jiang et al., 2014) of the EMG have been invested. In these machine learning approaches, the (correlated) EMG features of a larger number of electrodes is interpreted by an algorithm that is typically trained with user specific training data. However, despite several decades of research on these promising approaches, the majority of prostheses are still controlled with the conventional approach. Currently only one company offers an advanced, classification based controller, as an add-on for hand-prostheses of different manufacturers (Coapt, 2015). The main reason for this limited knowledge transfer from academia to clinics is the poor reliability of advanced myoelectric control methods when applied to real-world conditions (Jiang et al., 2012). Many of the factors that hinder reliability are related to the EMG electrodes, e.g., variations in the electrode position (Young et al., 2012) or changes in skin condition. Consequently, an advanced controller needs to be recalibrated frequently, in order to maintain an acceptable performance (Atzori and Müller, 2015).

In order to address some of the main reliability problems in machine learning based EMG control, in this work we present a novel type of electrode, fabricated with medical titanium and implanted percutaneously. With this implant reliability issues related to skin conditions, electrode lift-offs and variations of the electrode positioning are circumvented. These electrodes do not need wireless transmission but can be directly connected to external amplifiers. The surgery, which was successfully applied in four locations of the forearm of one able-bodied volunteer, is relatively simple and fast, with the level of invasiveness similar to regular cosmetic micro-dermal implants (Gaffaney, 2013). In this paper, we describe these new electrode implants and experimentally test their characteristics, as compared with those of two commonly used electrodes. The paper also reports results on simultaneous and proportional control (SPC) of multiple DoFs with the proposed electrodes.

## Methods

### Electrode design

**Figure 1:**
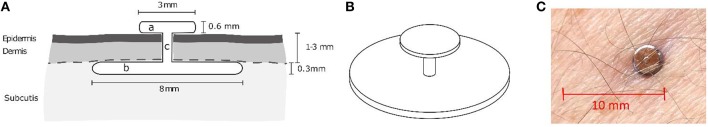
**Design and application of the implant. (A)** Schematic cross-section through the implantation site. **(B)** 3D-sketch of the implant. **(C)** Photo of an actual implant.

The one piece, passive electrode is fabricated from medical titanium, whose antimicrobial properties provide a good protection against infections. It consists of a subcutaneous disc for signal pickup, a thin linking axis for minimal skin penetration with length according to the thickness of the skin, and an epicutaneous part for external connection and sealing of the penetration (Figure 1).

### Implantation procedure

The electrode was implanted in four locations in the forearm of one of the authors (able-bodied man, 46 years). The self-experiment was reported to the local ethics committee who raised no objections.

The first implant was located in the distal part of the extensor digitorum muscle, approximately 1/3 of the distance from the wrist to the elbow. The other three implants were inserted 2 month later, after no complications were observed with the first one. They were located on the proximal portion of the extensor digitorum, flexor carpi radialis, and extensor carpi ulnaris muscles. The electrode positions were determined by palpation, and were intended to allow a myoelectric control task (discussed below). The thickness of the epidermis and dermis were identified by ultra-sound imaging and the implants were fabricated with customized length of the linking axis (section “c” in Figure 1A).

For implantation, incisions of approximately 10 mm in length were applied on the skin after localized anesthesia. The implants were inserted such that the larger disk was aligned below the dermis, the smaller disk above the epidermis, and the stem penetrated the skin. The wound surrounding the stem was closed by a single stitch suture. The wounds healed without complications for the four implants such that the skin closed around the linking axis of the implants after approximately 7 days, when the suture was removed.

### Evaluation procedure

The implants were tested for both their electrical properties as well as their applicability for myoelectric control. In these tests, the new electrodes were compared with classic disposable Ag/AgCl electrodes with conductive gel (Ambu Neuroline 720; pick-up area 95 mm^2^) and with dry electrodes commonly used in active electrode modules of myoelectric hand-prostheses (Otto Bock 13E200; pickup-area 40 mm^2^). The Ag/AgCl-electrodes and the dry electrodes were placed directly proximal and distal to the implants, because small displacements in the longitudinal direction with respect to the muscle fibers have a minor influence on the EMG characteristics (Young et al., 2012). The implants were connected with custom-made connection-clips which automatically disconnected in case the force applied was above a small threshold. The dry electrodes were fixed with medical tape and only the electrode-contacts closer to the implant were used, bypassing the integrated amplifier of the active electrode modules.

EMG signals were acquired with the same amplification, filtering, and acquisition chain for all the electrodes (the novel ones and those used for comparison). Signal amplification was obtained by a battery-powered, low-noise (<4 μV_RMS_) biosignal amplifier (OT-Bioelettronica USB-II) with a 16-channel pre-amplifier to minimize the influence of the cable length between the electrodes and the first amplification stage (approximately 10 cm for all electrodes). The sampling rate was 2048 Hz and the amplifier included a 3 Hz high-pass filter to remove DC offsets and a 900 Hz anti-aliasing low-pass filter. All signals were measured with reference the first implant. To compensate for the effect of this unbalanced reference (for Ag/AgCl and dry electrodes) and to reduce the influence of EMG-activity at the reference point, a group-wise common-mean referencing was performed individually for each electrode-type. To further reduce movement-artifacts, noise above the EMG-spectrum and power-line interferences including harmonics, a 4th order Butterworth-bandpass filter (30–500 Hz) and a comb-filter (50 Hz) were applied in software. The tests were conducted 3 month after the implantation of the last implants.

#### Impedance measurements

The electrical impedance is an important characteristic of the electrode-tissue interface (Merletti and Parker, 2004). Desirable characteristics of electrode-tissue impedance are a low value of the impedance and a balance of values between the electrodes used for a differential derivation (Merletti et al., 2009). Clinically available myoelectric hand prostheses typically need some time after placement before they would work optimally, because the electrode-skin impedances of the dry electrodes gradually decrease after placement until the background noise is below a certain value. High impedances (e.g., caused by dry skin) can significantly decrease the robustness of the control and the sensitivity to artifacts under conditions of daily use of a prosthesis. As myoelectric control techniques can be influenced by a variation of the noise-level, a stable impedance over time is beneficial.

The electrode impedance was measured over a frequency-range of 0–900 Hz, using a current of 500 μA. To investigate inter-day variations in the impedance of the implants, the measure of impedance was repeated over 3 days (day one, two, and five). For the dry and the wet Ag/AgCl electrodes, the impedance was measured over time at 3, 10, 45, and 120 min following placement.

Three of the implants were used in the myoelectric control test against the first implant as reference point. All other electrodes were tested against one wet Ag/AgCl electrode close to the implant reference. To correct for the impedance of the reference electrode, this was estimated to be half of the average of the impedance measured from all the electrodes within the same type (Ag/AgCl, implants). The measurement resolution was 100 Ω and all measurements were repeated three times (of which we report the average values).

#### Signal quality and mechanical interference

To qualitatively compare the signals acquired by the different electrode types, a series of wrist-extensions between 10 and 50% maximum voluntary contraction (MVC) force, were performed and the EMG signals were recorded concurrently with all three electrode-types. The forearm was rested on a comfortable arm-rest and the force was measured by a strain gauge which was calibrated relative to 100% MVC. Ten contractions of 5 s duration were recorded per force level, interleaved with periods of 5 s of rest in between the contractions. The amplitude and the frequency spectra were analyzed and the SNRs were estimated by the ratio of the root mean square amplitude of the signals during the contraction phase and during rest for intervals of 3 s. This estimate assumes that the noise level is relatively small so that the root mean square during the contraction can be approximately considered as not influenced by noise.

In myoelectric prostheses, the mechanical interference transmitted to the socket and to the integrated electrodes is an important source of signal noise. Therefore, a test was performed for systematically comparing the sensitivity of the electrodes to mechanical vibrations. For this purpose, EMG signals were recorded while the subject used a pneumatic impact driver which delivered strong mechanical vibrations to the forearm. Alternating phases of 15 s mechanical vibrations and 15 s of pauses were applied to the forearm while the subject was asked not to activate his muscles.

#### Real-time myoelectric control task

The performance of myoelectric control algorithms degrades significantly over days without a re-training of the system. This is largely due to the repositioning of the electrodes when donning/doffing the prosthesis (Young et al., 2012). A myoelectric control test that involved simultaneous control of two DoF of the wrist was designed based on the linear regression approach described in Hahne et al. (2015). The wrist angles of flexion/extension and radial/ulnar deviation were estimated from the RMS of the EMG in overlapping time intervals of 200 ms, with 40 ms increments. To train the regressor, EMG was recorded simultaneously with all three electrode types, while the subject followed visual cues on a computer screen. Then three linear regressors were trained, each based on the EMG signals of one electrode-type. The visual cues involved three repetitions of all four directions and formed the labels y, which were required for the supervised training procedure of the linear regression models W. This procedure excluded the human factor from the training procedure as the signals of all regressors where obtained from identical trials.

**Figure 2:**
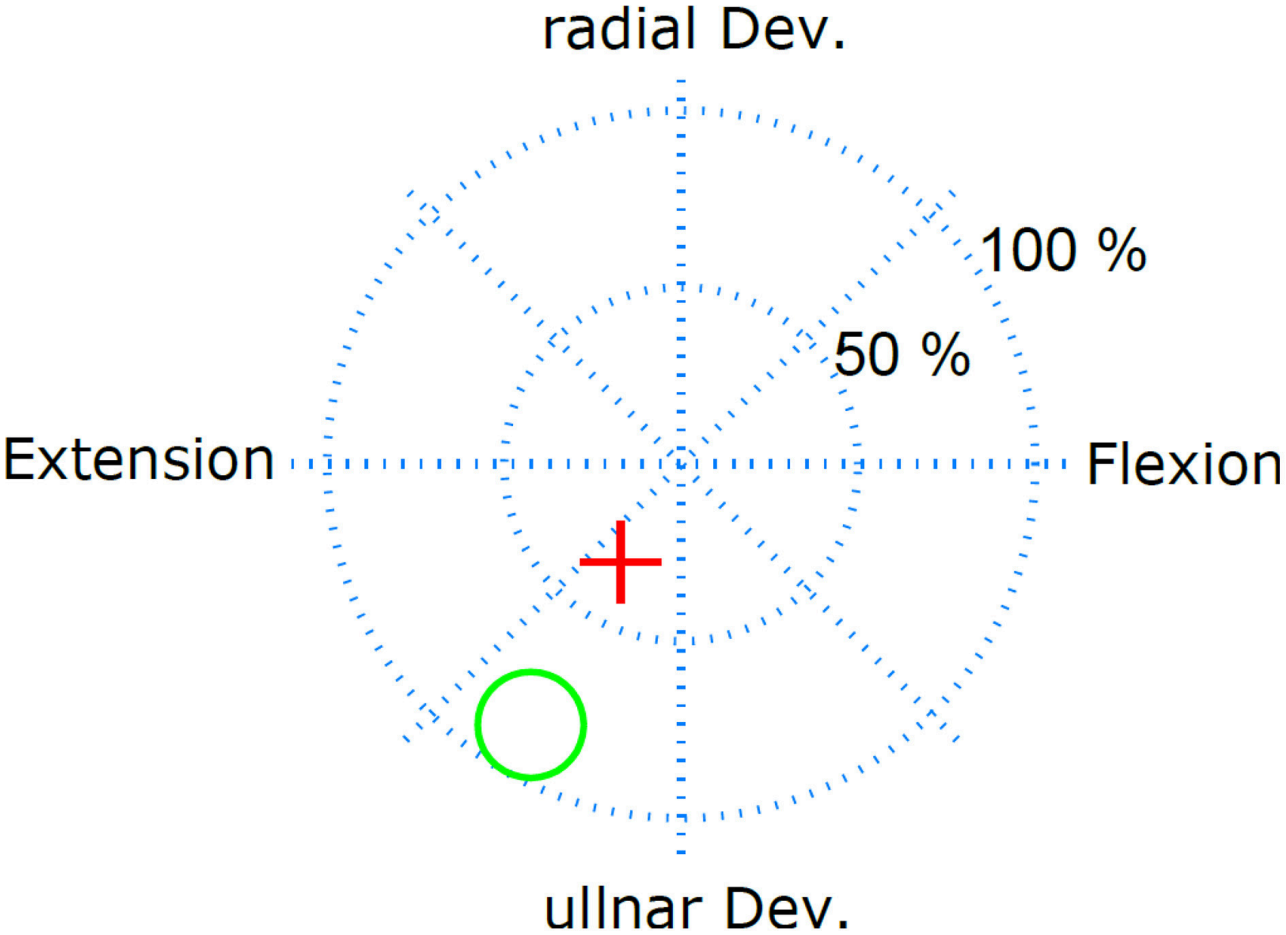
**Real-time myoelctric control task**. The subject controlled the red cursor with his EMG and had the task to hit the target (green circle) and remain in the target for 1 s. The subject had 10 s to perform this task.

The performance was evaluated in real-time control tasks, in which the two dimensional output of the linear regressor was used to control the position of a cursor in 2D (Figure 2). Specifically, the subject was asked to hit circular targets by moving the cursor into the target and remaining in the target for 1 s, before a 10 s time-out. The test was repeated over 2 days, twice per day, without re-training. The order in which the electrodes were tested was randomized and blind to the subject. To minimize the shifts over days of the classic electrodes, the position of the two classic electrodes was marked on the skin on the first day. The performance was quantified with two metrics: task completion rate and task completion time. The task completion rate is defined as the ratio of targets that were successfully hit before time-out and the total number of targets presented. The task completion time is the average time spent per target.

## Results

### Impedance

**Figure 3:**
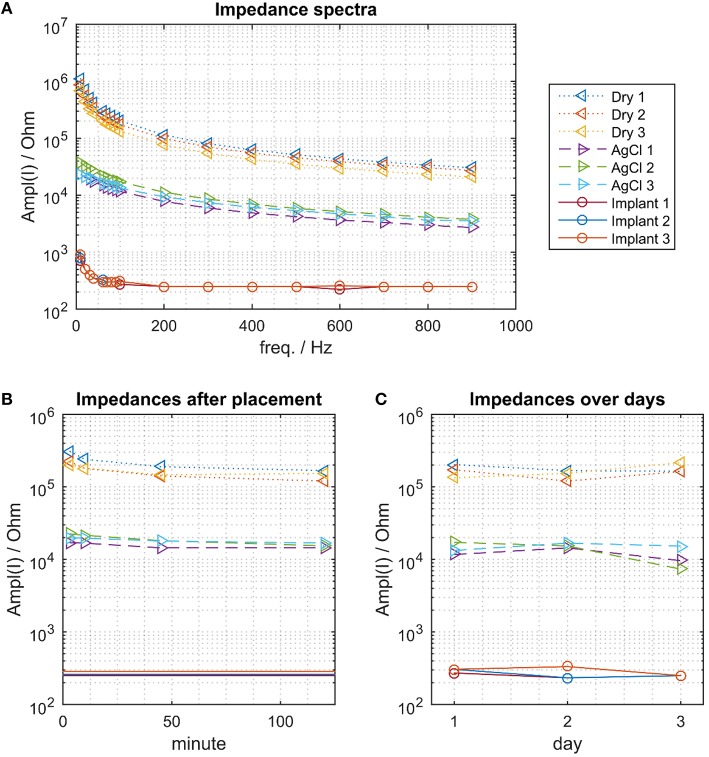
**The electrical impedance for the 3 electrode types. (A)** Impedance spectra. The proposed implant has the smallest impedance, followed by the wet AgCl electrodes and the dry electrodes. **(B)** Impedance change following donning (at *f* = 100 Hz). The impedance of the dry electrodes decreased continuously within the 120 min of measurements. This effect was less pronounced for the Ag/AgCl electrodes. **(C)** Impedance variations across days, measured approximately 120 min after placing the dry and Ag/AgCl electrodes.

As common for the electrode-tissue-interface, the impedance decreased with increasing frequency for all electrode types (Figure 3A). While the impedance spectra had little between-session variability for each type of electrode, the impedance- spectra of the 3 types of electrode were different. The dry electrodes showed the largest impedance, the Ag/AgCl electrode had moderate impedance and the proposed implants the smallest impedance (by more than two orders of magnitude for the dry electrodes and one order of magnitude for the AgCl electrodes). At *f* = 100 Hz, which is approximately in the center of the EMG bandwidth, the dry electrodes had an impedance of approximately 100–200 kΩ, the Ag/AgCl electrodes of approximately 10–20 kΩ, and the implants of approximately 200–300 Ω.

The impedance of the dry electrodes asymptotically decreased after donning and approximately halved over a time interval of 120 min (Figure 3B). As expected, such a decreasing trend was less pronounced in the Ag/AgCl electrodes. As the implants were chronically implanted, this transient did not exist and the impedance of the implants remained stable across days (Figure 3C). When waiting 120 min following the electrode montage, the impedance of the dry and AgCl electrodes was also stable across days (Figure 3C).

### Signal quality and robustness against mechanical interference

**Figure 4:**
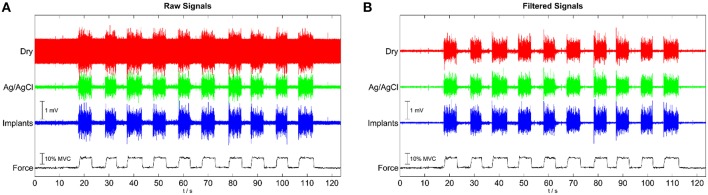
**Representative EMG signals for contractions with 10 % MVC, simultaneously recorded with the implants, wet, disposable Ag/AgCl electrodes and dry metal electrodes. (A)** raw signals; **(B)** after common mean subtraction and temporal filtering.

**Figure 5:**
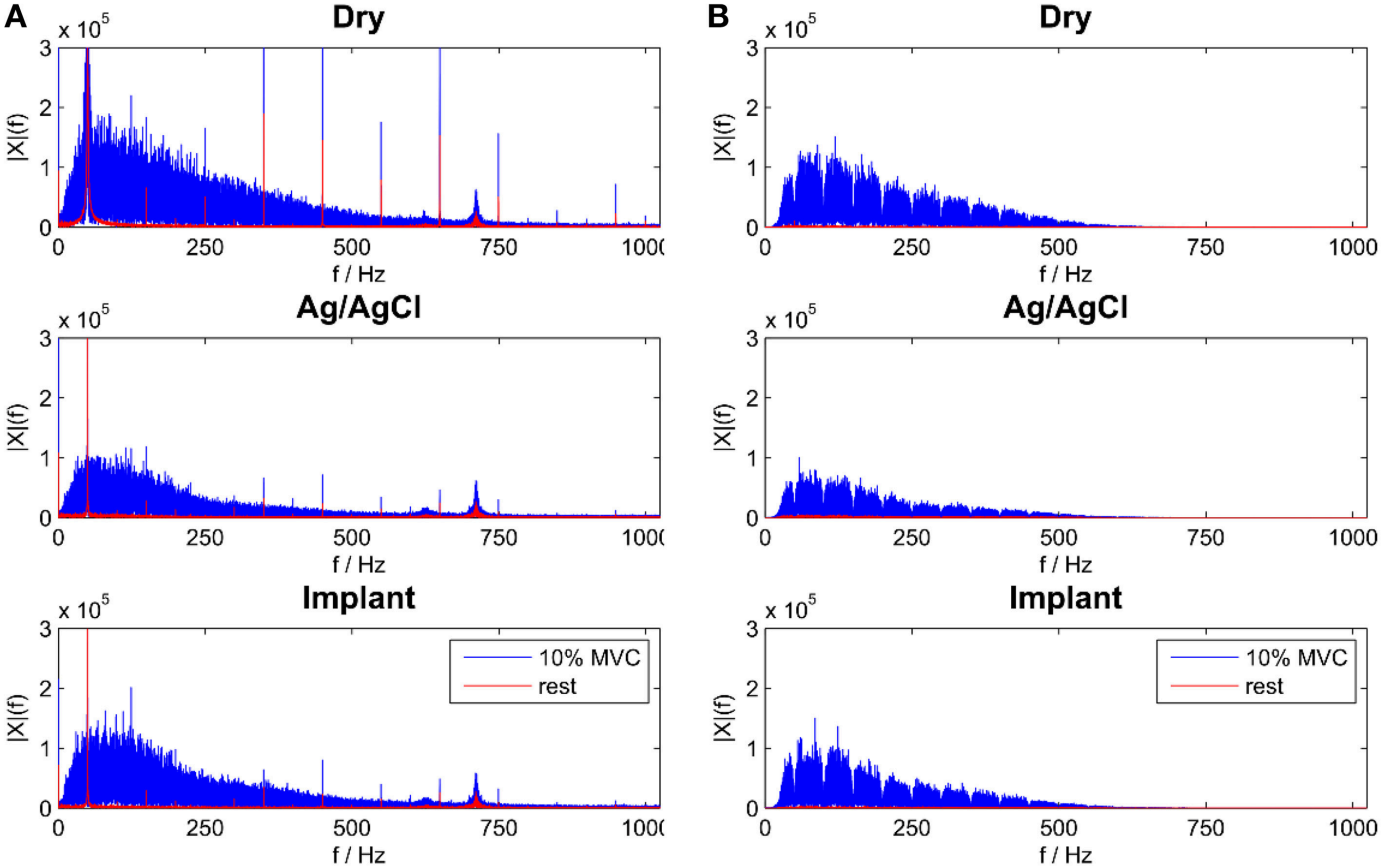
**Frequency Spectra for all electrode types during rest and 10% MVC contraction for raw (A) and temporal filtered signals (B)**.

**Figure 6:**
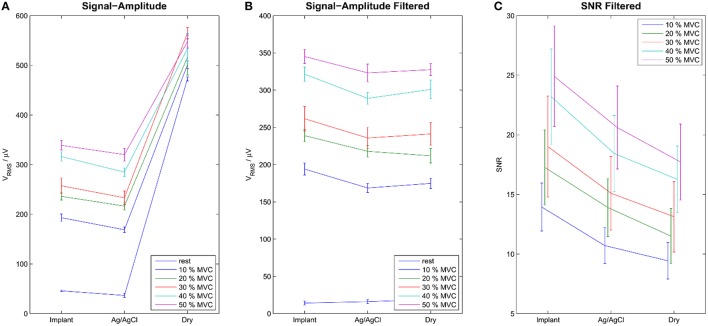
**Signal Amplitudes of the raw (A) and the filtered (B) signals during rest and contraction of various levels**. Error-bars show inter-trial standard deviation. SNR is shown only for the filtered signals **(C)**. Without filtering the noise level was very high, exceeding the level of EMG signal for dry electrodes. After temporal filtering and common mean subtraction, the difference between the electrode types was less pronounced. The Implants showed the best SNRs, followed by the gelled Ag/AgCl and the dry electrodes.

The signal quality of the tested electrode types was evaluated in the time (Figure 4) and frequency domains (Figure 5). Without filtering, the signals were strongly corrupted by noise. For the dry electrodes, the baseline noise reached levels of approximately 500 μV_RMS_, which was greater than the amplitude of the EMG signals. The frequency spectra revealed that most of the noise was due to powerline interference at 50 Hz and its higher order harmonics. After filtering, all electrodes-types had baseline noise levels <20 μV_RMS_. With signal amplitudes in the range of 180–350 μV_RMS_, depending on the contraction force and electrode type, this corresponded to an SNR between 10 and 15 for 10% MVC and 18–25 for 50% MVC (Figure 6). Among the investigated electrode types, the implants had the best SNR followed by the gelled Ag/AgCl and the dry electrodes.

**Figure 7:**
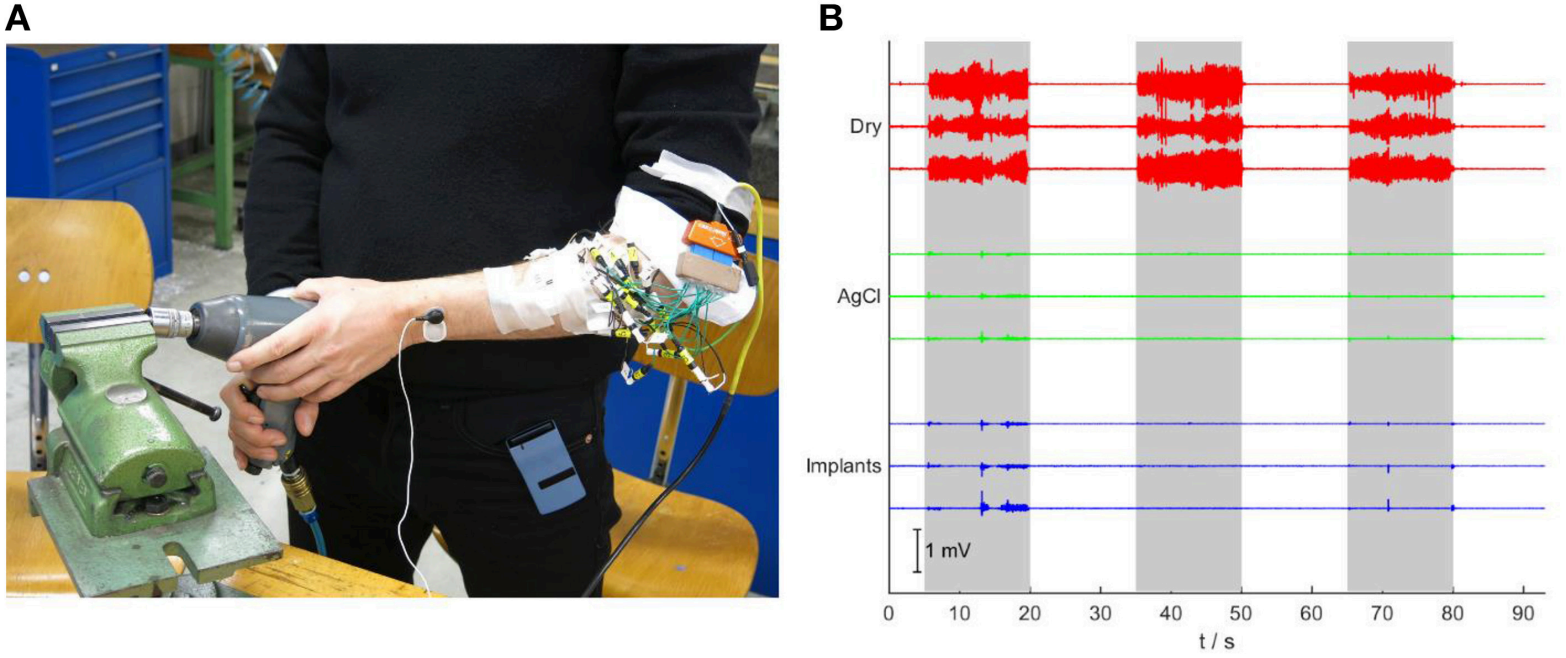
**Robustness test against mechanical vibrations. (A)** The subject is using an impact driver while signals are acquired. **(B)** Signals recorded during mechanically applied vibrations to the forearm. While the signals of the dry electrodes show large artifacts during the three periods in which the vibrations are applied (gray background), the signals of the gelled Ag/AgCl electrodes and the implants are almost not affected.

In the test with mechanical interference (Figure 7A), the dry electrodes showed strong artifacts during the interference periods, with a broad frequency spectrum between 0 and 100 Hz (peak below 20 Hz). Even after band-pass filtering (30–500 Hz), the amplitude of the artifacts exceeded those of the EMG signals during moderate contractions (Figure 7B). The Ag/AgCl electrodes and the implants were significantly less influenced by this type of interference. The small activity detected by those electrodes (mainly in the interval 13–20 s) is presumably caused by stabilizing muscle contractions, as an involuntary response to the mechanical interference.

### Myoelectric control task

**Figure 8:**
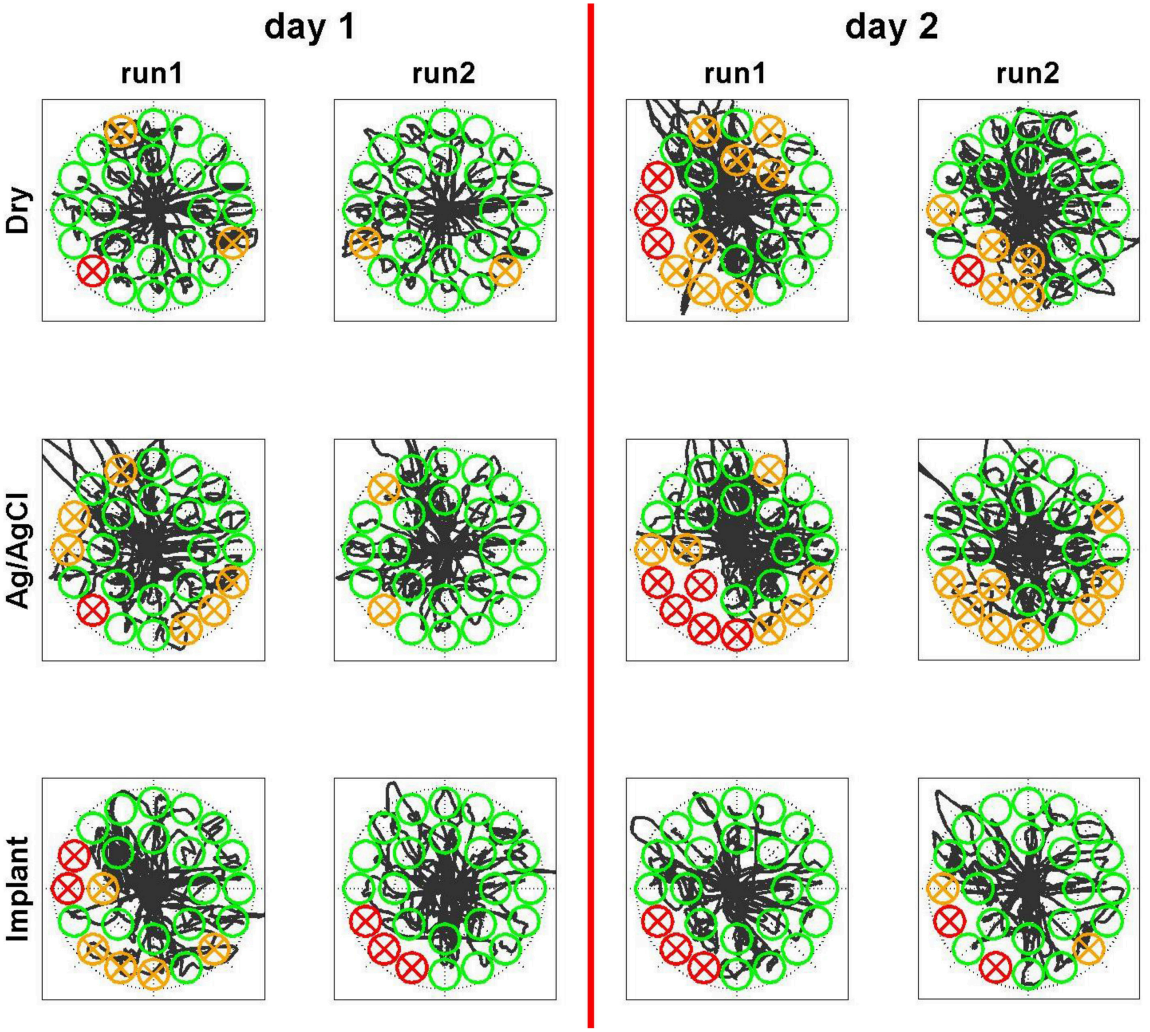
**Qualitative perfromance evaluation in Myoelectric control task**. The subject controlled simultaneously both dimensions of a cursor with his EMG. He had the task to steer the cursor into the randomly appearing circles and remain there for one second. The linear control model was trained on the first day before run 1. Sucessfully hit targets are shown in green, missed targets in red and targets that where entered but not hit due to insufficient dwell time are shown in orange. The black curve indicates the trace of the cursor. While the performace drops on the second day for both surface electrode types, it remains stable for the implants even across days without retraining the model.

**Figure 9:**
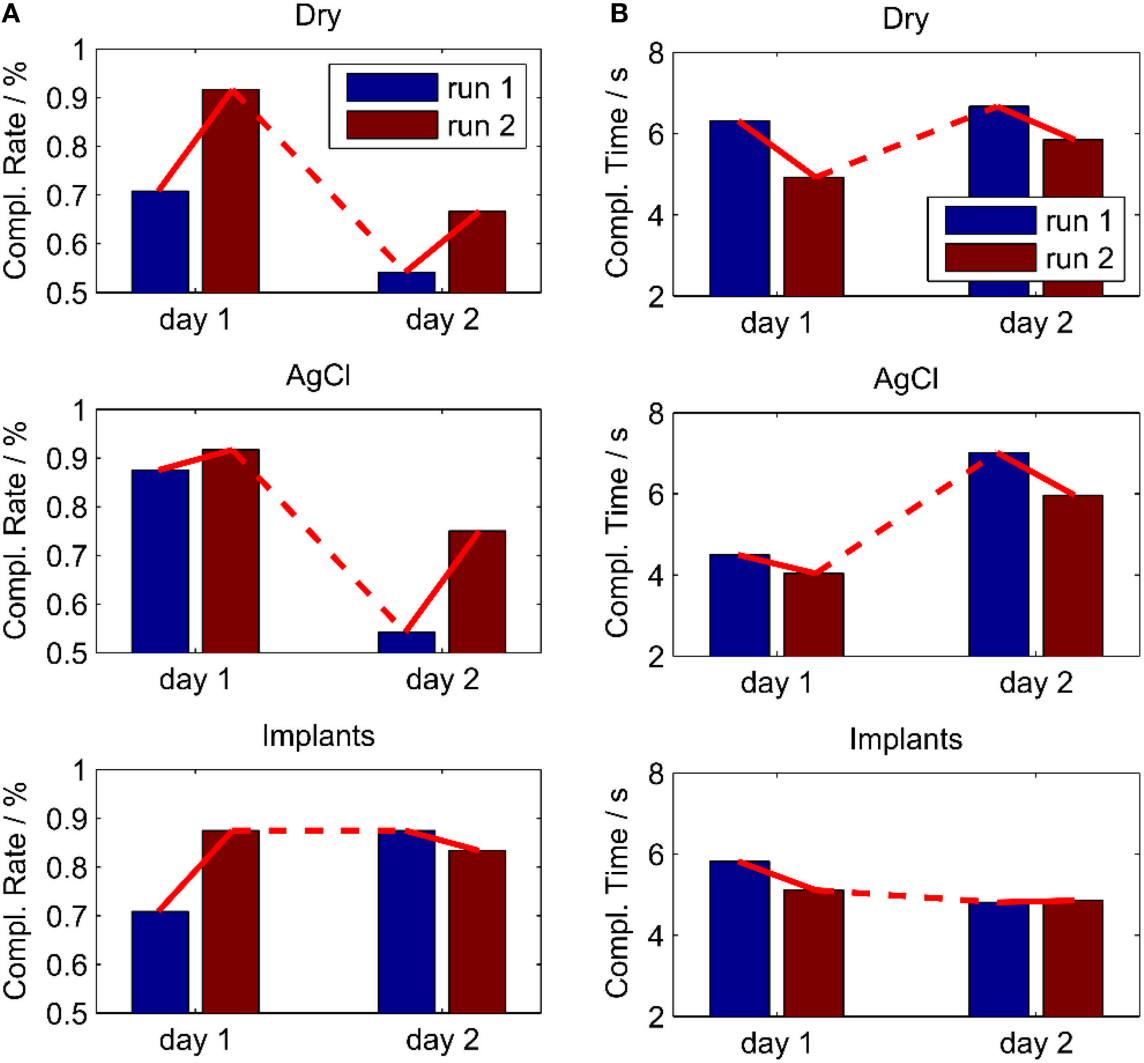
**Performance in an online myoelectric control task, performed twice for all electrodes on two different days**. The control algorithm was calibrated in day 1 and used also in day 2 without re-calibration. **(A)** Completion Rate; **(B)** Completion Time. With the implants, the performance remained stable over the 2 days contrary to the other electrode types.

After the short calibration phase based on visual cues, the subject was able to reliably control the cursor on a computer screen with all three electrode types. The control performance increased between the first and the second run on the first day for all electrode types, which was presumably due to learning effects of the subject. The relatively straight cursor paths in run 2 indicate that the subject had a very good controllability of the cursor with his EMG (Figure 8). In the second run of day 1, for all electrode types, the task completion rates were ~90% and the task completion time was 4–5 s per target (Figure 9). When testing the same regression models on the 2nd day, the performance obtained with the implanted electrodes remained stable, whereas it dropped substantially for the two classic electrodes. Certain areas in 2D where not accessible anymore, while the subject overshot for other directions. These problems were only partly compensated in the second run of day 2, likely due to user adaptation.

## Discussion

We developed a novel, per-cutaneous electrode Implant to acquire stable EMG signals for a potential application in prosthetic control, with the aim to solve reliability problems that come along with surface EMG. Alternatives to the proposed systems are intramuscular (Weir et al., 2009) and epimysial (Lewis et al., 2013) electrodes. These approaches acquire the signals closer to the origin but require a more complicated surgery. Additionally, elaborated and costly electronics, inductive power-transmissions and RF links to transmit the data are needed. Our system is much simpler and cheaper to produce.

The proposed implant showed very good electrical characteristics. The impedance was substantially lower than classic electrodes and remained stable across days. The noise level was similar as that of gelled Ag/AgCl electrodes, which can be seen as a gold standard for acute EMG recordings (not applicable for prosthetics). Dry electrodes, which are commonly used in prosthetics, had impedance that was almost three orders of magnitudes greater than the proposed implant and resulted in very large noise levels. Appropriate filtering techniques could reduce the noise level, but the dry electrodes were still outperformed by the implants even with filtering. Moreover, the dry electrodes reacted strongly on mechanical interference, which would make a prosthesis not usable with large vibrations. The implants on the other hand were not influenced by these interferences.

Other implants for myoelectric control have been proposed earlier. The IMES (Weir et al., 2009; Pasquina et al., 2015) is a very promising approach in which miniaturized EMG amplifiers with a telemetry unit are implanted directly into multiple muscles. With this technology also deep muscles can be accessed and due to the high selectivity the concept of direct control can be extended to multiple degrees of freedom (parallel dual-site differential control; Smith et al., 2014). Machine learning based approaches do not require such a high specificity as the parallel dual-side control, but stable signal patterns over time to avoid recalibration. Another promising approach based on an implantable, flexible electrode array has been proposed by Lewis et al. (2013). Both approaches have the advantage that no link through the skin remains after implantation. However, they require sophisticated electronics including telemetry and inductive power transmission with limited power efficiency. This would increase the costs and the power consumption of a prosthesis. Our approach is significantly simpler. In case of failure of the electronics, an external amplification unit could be exchanged without the need to change the implant. Also a removal of the device is significantly easier than that of deeply implanted devices.

The clearest results on the advantage of the proposed implant are from the real-time myoelectric control for the regression based simultaneous and proportional control of two DoFs. After a short training period, the subject achieved a very good controllability with all three electrode types. However, when the regression model of the first day was applied on a second day, the performance dropped substantially for the dry electrodes and the Ag/AgCl electrodes. In a prosthetic application, this would require a re-training of the algorithm, a significant daily effort, which is a major user-compliant issue. The proposed implant showed stable results across days and would therefore avoid the need for re-training in a prosthetic application. The lack of reliability is one of the most important problems in machine learning based techniques for myoelectric control and it is the main reason for the limited transfer of academic achievements in the past decades into clinical practice. Small shifts in the electrode position and changing of skin conditions are two of the most important factors that degrade the performance under real-world conditions (Jiang et al., 2012). Our findings indicate that the problems related to both factors may be solved by the proposed implants. We have recently shown that with appropriate training strategies prosthetic users can achieve a similar high performance as able-bodied subjects in regression based simultaneous and proportional myoelectric control (Hahne et al., 2015). Once a high performance is reached, we expect a similar robustness to the investigated factors also for prosthetic users with the proposed implants. The implant could also be used in combination with hybrid control systems, in which different signals, such as EMG and inertial sensors, or different control strategies, such as regression and classification are combined.

It has been shown for a classification based approach that the performance, obtained with intramuscular EMG is similar to that of surface EMG, but targeted placement is required for intramuscular EMG due to the very high specificity (Farrell and Weir, 2008). As in our approach mainly superficial muscles are targeted, it is in some aspects similar to surface EMG. However, the spatial blurring effect and the additional impedance of the skin as well as position and skin-condition related reliability problems are circumvented.

Even though the tolerance of the device to mechanical stress was not explicitly evaluated in this study, we expect a similar tolerance as for cosmetic micro-dermal implants. It remains to be evaluated how the device would behave within a prosthetic socket. In a future application, cavities in the inner prosthetic socket could prevent a direct mechanical contact between the socket and the implant during use. In a future design, the connectors could attach and detach automatically with flexible magnetic connectors. With this connection technology the proposed implant would be also robust against electrode lift-offs that remain a problematic issue in conventional dry electrodes, especially when the volume of the residual limb is changing.

In general, the potential risk of infection is another key user-compliant issue for any implant. Our approach has the disadvantage of a permanent link through the skin with potential risks of infection. However, the link through the skin has a diameter of only 0.5 mm and due to the individual adjustment to the thickness of the skin, an antibacterial sealing by the internal and external disks is achieved. Thus, the infection risk of the proposed implant is expected to be not higher than that of frequently used cosmetic micro-dermal implants. To fully prove issues such as safety and increase the statistical reliability of the results, a full clinical evaluation with a larger number of subjects would be required. Also an evaluation with prosthetic users controlling their prostheses with the proposed implant and advanced control strategies should be conducted in the future.

## Conclusion

We have proposed a novel implanted electrode for myoelectric control that does not require wireless transmission of information or powering as needed in previous approaches (Weir et al., 2009; Lewis et al., 2013) and that requires a minimally invasive surgical procedure. The electrode showed advantages over the surface electrodes currently used for EMG detection, including those applied for control in commercial prostheses (the dry electrode type). The new electrode had superior electrical properties and was less influenced by mechanical interference than those used in active prostheses. Moreover, the implants showed a stable myoelectric control performance even when tested on a second day without retraining of the control algorithm.

In conclusion, the proposed implants may be a promising interface for current commercial and clinical prostheses.

## Author contributions

Development and Implantation of the Device: DL Design of the Study and the Experiments: JH, DF, DL, NJ. Executing the Experiments: JH, DL, NJ. Writing Manuscript: JH. Revising Manuscript: DF, NJ, DL.

### Conflict of interest statement

The authors declare that the research was conducted in the absence of any commercial or financial relationships that could be construed as a potential conflict of interest. The reviewer LH and the handling Editor declared their shared affiliation, and the handling Editor states that the process nevertheless met the standards of a fair and objective review.
